# Supplementary material for: A Bioinformatics Pipeline to Identify a Subset of SNPs for Genomics-Assisted Potato Breeding
Source: Plants (Basel). 2020 Dec 24;10(1):30. doi: 10.3390/plants10010030 (PMC7824009; doi:10.3390/plants10010030)
Supplement: Supplementary file 1 [file plants-10-00030-s001.zip › supplementary materials/table-S1.docx]

**Supplementary table S1**

| **Heritability (H²) and variance components (VAR)** | | | |
| --- | --- | --- | --- |
| Source of variation | AUDPC | TN | TW |
| Block error (adj.) | 3070 | 16 | 0.23 |
| Intra block error | 2311 | 20 | 0.09 |
| Genotypes | 25743 | 144 | 1.88 |
| VAR (genotypes) | 23257 | 125 | 1.76 |
| VAR (phenotypes) | 26365 | 149 | 1.91 |
| H² | 0.88 | 0.84 | 0.92 |
| Fc MS Error | 1.34 | 0.83 | 2.56 |
| P(Fc > Ft) | 0.79 NS | 0.50 NS | 0.99 ** |
