# Supplementary figures and images for: A Bioinformatics Pipeline to Identify a Subset of SNPs for Genomics-Assisted Potato Breeding
Source: Plants (Basel). 2020 Dec 24;10(1):30. doi: 10.3390/plants10010030 (PMC7824009; doi:10.3390/plants10010030)

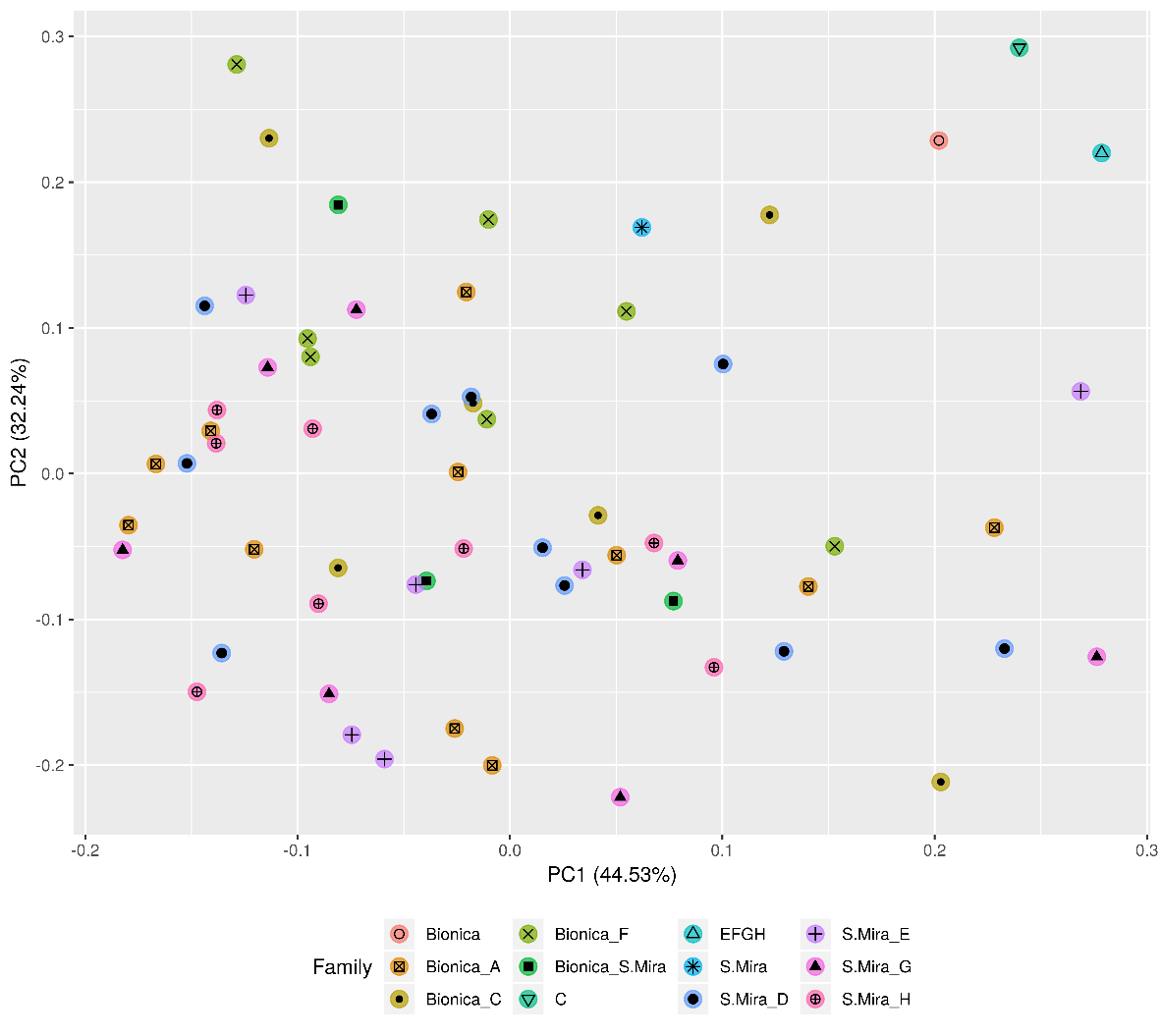


**Supplementary Figure 1**

Supplement: Supplementary file 1 [file plants-10-00030-s001.zip › supplementary materials/figure-S1.docx]

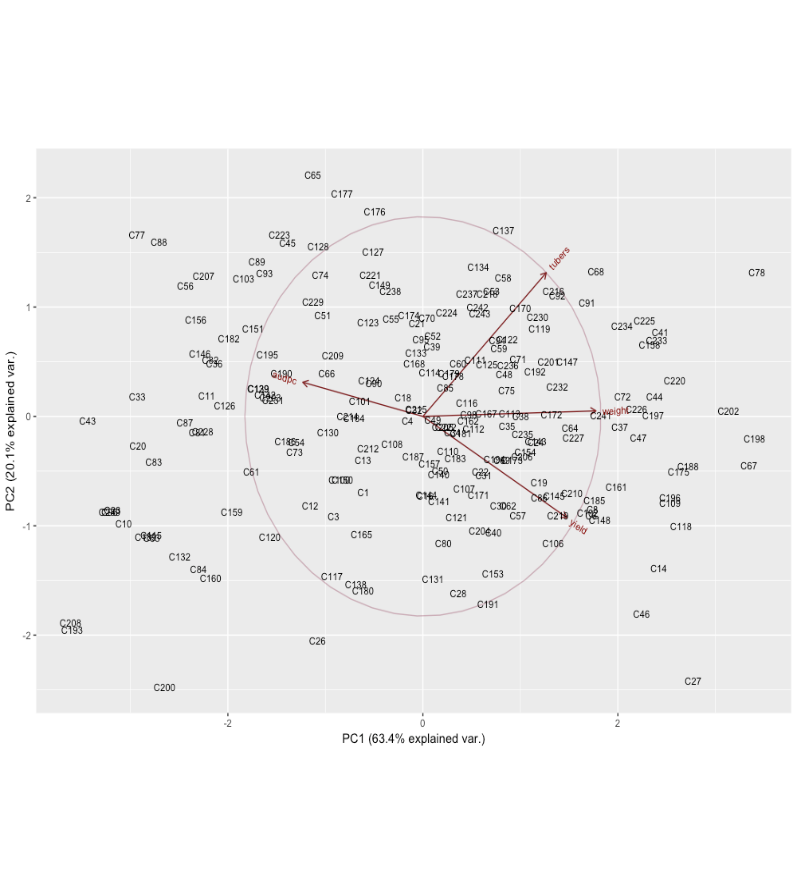


**Supplementary Figure 2.**

Supplement: Supplementary file 1 [file plants-10-00030-s001.zip › supplementary materials/figure-S2.docx]
